# Downregulation of microRNA-17-5p improves cardiac function after myocardial infarction via attenuation of apoptosis in endothelial cells

**DOI:** 10.1007/s00438-018-1426-5

**Published:** 2018-03-13

**Authors:** Shuo Yang, Tao Fan, Qi Hu, Weipan Xu, Jian Yang, Changwu Xu, Bofang Zhang, Jing Chen, Hong Jiang

**Affiliations:** 1 Department of Cardiology, Renmin Hospital of Wuhan University, Cardiovascular Research Institute of Wuhan University, 238 JieFang Road, Wuhan, 430060 China; 2 Department of Thoracic Surgery, Remin Hospital of Wuhan University, Wuhan, China; 3 Department of Cardiology, Huangshi Central Hospital, Huangshi, China; 4 Department of Cardiology, Yichang Central People’s Hospital, Yichang, China

**Keywords:** MicroRNA-17-5p, Endothelial cells, Apoptosis, Myocardial infarction

## Abstract

MicroRNA-17-5p (miR-17-5p) was indicated to suppress the formation of blood vessels, which is associated with cardiac function after myocardial infarction. In this study, the relationship between miR-17-5p and cardiac function was researched. Human umbilical vein endothelial cells were infected with adenoviruses. Apoptosis was determined by Annexin V-7AAD/PI. Real-time RT-PCR was used to evaluate miR-17-5p and ERK levels. Western blotting was used to determine the levels of ERK, the anti-apoptosis protein bcl-2 and apoptosis proteins, including bax, caspase 3, and caspase 9. An in vivo acute myocardial infarction (AMI) model was established in SD male rats. Heart function was evaluated by echocardiography prior to inducing AMI and after 7 and 28 days later. The heart was removed to perform histological examination, real-time RT-PCR, and western blotting, as described above. The result indicated that the ERK pathway was activated by miR-17-5p downregulation and an increase in the level of the anti-apoptosis protein bcl-2; however, the levels of apoptosis proteins (bax/caspase 3/caspase 9) were decreased. The results were completely reversed when miR-17-5p was up-regulated. At 7 and 28 days after the induction of AMI, in the miR-17-5p inhibition group, the infarction areas and collagen fibers were decreased, apoptosis in cardiac tissues was inhibited, and the endothelial growth process was promoted. Therefore, MiR-17-5p silencing protects heart function after AMI through decreasing the rate of apoptosis and repairing vascular injury.

## Introduction

Myocardial infarction is one of the leading causes of death globally and is mainly characterized by vascular endothelial injury due to apoptosis, cardiac fibrosis, inflammation, and pathological remodeling (Du et al. 2014). Though precautions and medical treatments for myocardial infarction have improved, a considerable number of deaths due to myocardial infarction occur every year (Zhang et al. 2012). Heart failure after myocardial infarction (MI) is closely associated with microcirculation (Al Suwaidi et al. 2000; Joner et al. 2006; Farb and Boam 2007; Dangas et al. 2010). Studies have confirmed that the common pathological physiological basis of myocardial infarction is delayed repair of the damaged vascular endothelium (Finn et al. 2007; Inoue et al. 2011). It is highly important to completely restore endothelial function soon after myocardial infarction.

MicroRNA is a newly discovered class of small non-protein-coding mRNAs, which have rapidly became known as critical regulators of gene expression (Krol et al. 2010). Mature microRNAs negatively regulate gene expression through two mechanisms: they prevent protein synthesis at the translational level and cause degradation of the mRNA of the target gene (van Rooij 2011). Every microRNA can control the expression of multiple genes simultaneously, which makes the translation of traditional single locus control to multiple site intervention possible (van Rooij 2011). Cell-specific expression is another major characteristic of microRNAs (van Rooij 2011). Through post-transcriptional regulation, microRNAs define the proteomes of cells by regulating gene expression, and their regulatory activities are involved in various cellular functions and generate specific biological phenotypes (Norbury 2010). Recently, many miRNAs have been confirmed to be involved in the regulation of the pathological and physiological processes of heart disease (Ren et al. 2009; Wang et al. 2010; Pan et al. 2012).

Researchers have shown that microRNA-17 (miR-17) is highly expressed in endothelial cells (EC) (Bonauer et al. 2009) and has low expression in vascular smooth muscle cells (VSMC). Researchers have also found that miR-17 exerts an anti-angiogenic function; therefore, inhibiting its expression can improve the density of EC angiogenesis in vitro (Doebele et al. 2010; Yu et al. 2010). Other studies have indicated that in the process of rat carotid artery injury and repair, the expression of miR-17 rises continuously, while the expression of miR-18a and miR-19a, which are known to promote angiogenesis, show no obvious changes (Ji et al. 2007). Here, we hypothesized that downregulation of miR-17 would enhance cardiac function after MI by improving endothelial function and increasing the density of new blood vessels.

## Materials and methods

### Cell culture and adenovirus infection

Human umbilical vein endothelial cells (HUVECs) were purchased from Sciencell Company (Cat. No. 8000) and cultured with endothelial cell medium (ECM) (Sciencell Cat. No. 1001) containing 5% fetal bovine serum (FBS), 1% endothelial cell growth supplement (ECGS), and 1% penicillin/streptomycin solution (P/S). All HUVECs were used for experiments between the first and fifth passages. Recombinant adenoviruses containing pre-miR-17 or Antagomir-17 for miR-17-5p overexpression (Ad-Pre-miR-17) and miR-17-5p silencing (Ad-Antagomir-17), respectively, were prepared. Ad-NC-Pre-miR-17, which contains the same gene sequence as Pre-miR-17, but does not affect the expression of miR-17-5p, was used as the control adenovirus for Ad-Pre-miR-17. Ad-NC-Antagomir-17, which contains the same gene sequence as Antagomir-17, but does not affect the expression of miR-17-5p, was used as the control adenovirus for Ad-Antagomir-17. For adenovirus infection, the above-mentioned HUVECs were incubated with Ad-Pre-miR-17/Ad-NC-Pre-miR-17 or Ad-Antagomir-17/Ad-NC-Antagomir-17 at a multiplicity of infection (MOI) of 30 in serum-free media for approximately 4 h. Then, the media were removed, and the cells were incubated with complete medium for the next 72 h.

### Apoptosis analysis

Cell apoptosis was measured in an Annexin V-7AAD/propidium iodide (PI) assay. HUVECs infected with the recombinant adenoviruses mentioned above were divided into three parts and seeded in 60-mm plates (1 × 10^5^). After synchronization in ECM with 0.5% FBS for 24 h, one part was cultivated in ECM containing 5% FBS for 24 h, and the second part underwent apoptosis in ECM without FBS for 24 h, and the third part treated with ERK inhibitor PD098059 at a concentration of 10 µM in ECM without FBS for 24 h. Cells were collected and re-suspended in 1× binding buffer. Apoptotic cells were measured with an Annexin V-7AAD apoptosis detection kit (BD Biosciences, USA) according to the manufacturer’s protocol. Five microliters of Annexin V-7AAD and 5 µl of PI were added to the re-suspended cells, and the cells were incubated for 15 min at room temperature in the dark. Apoptosis was measured using a BD accuriC6 flow cytometer, and the data were processed using FlowJo (FlowJo, Ashland, OR, USA) software.

### Real-time RT-PCR

Total RNA was extracted from cells or hearts with Trizol reagent (Invitrogen), then reversely transcribed to cDNA using Quantscript RT kit (Tiangen, Beijing, China). U6 and mRNAs were converted using random hexamers, while miR-17 was reversely transcribed using gene-specific primer. qPCR was performed in triplicates using SYBR Premix Ex TaqTM II (TaKaRa, Japan) on Step-one plus real-time PCR system (Applied Biosystems, Foster City, CA, USA). GAPDH (for mRNAs) and U6 (for microRNAs) were used as internal controls for normalization and gene expression level was calculated using 2-ΔΔCt method. The primer sequences were as follows:

miR-17-5p Forward primer: 5′GGGGCAAAGTGCTTACAGTG3′

Reverse primer: 5′GTGCGTGTCGTGGAGTCG3′

U6 Forward primer: 5′GCTTCGGCAGCACATATACTAAAAT3′

Reverse primer: 5′CGCTTCACGAATTTGCGTGTCAT3′

ERK Forward primer: 5′GAGGTTGACCACGGTGGAAT3′

Reverse primer: 5′ TTTGGTTTCCCACGGCTTCT3′

GAPDH Forward primer: 5′GGGAAACTGTGGCGTGAT3′

Reverse primer: 5′GAGTGGGTGTCGCTGTTGA3′

### Western blotting

For whole-cell protein extraction, HUVECs under apoptosis induction were washed three times with cold PBS and subsequently lysed in cold RIPA lysis buffer (20 mM Tris Cl, pH 7.4/137 mM, NaCl/2 mM EDTA/1% Triton/10% glycerol/25 mM β-glycerol phosphate/1 mM PMSF, and protease inhibitor mixture). Cell or heart lysis was performed on ice for 30 min, and clear protein extracts were obtained by centrifugation at 12,000×*g* for 10 min. Protein concentration was determined with the bicinchoninic acid protein assay (Bipec). Proteins were separated by SDS–polyacrylamide gel electrophoresis and were transferred to PVDF membranes. For immunoblotting, PVDF membranes were blocked with 5% milk and probed with antibodies against total ERK and phospho-ERK, bax, bcl-2, caspase 3, caspase 9 or GAPDH (all from Abcam) overnight at 4 °C. After washing the membranes with TBST three times (10 min each time), they were incubated with peroxidase-conjugated secondary antibodies (BOSTER) for 2 h at room temperature. The proteins were detected using the ECL Plus detection kit (Bipec).

### SD rat AMI model

All animals used in this study were provided and cared for by the Animal Center of Renmin Hospital of Wuhan University. The experimental procedures and animal care were approved by the Animal Care and Use Committee of Wuhan University. All animals were given a conventional diet until they were sacrificed. All of the animal protocols complied strictly with the Institutional Animal Care and Use Committee guidelines. The procedure for inducing myocardial infarction (MI) in rats was described previously. Briefly, male Sprague–Dawley (SD) rats (200–250 g) were anesthetized with pentobarbital (30 mg/kg intraperitoneal injection). The adenoviruses (5 × 10^9^/100 μL) mentioned above were injected into the apex of the heart after the left anterior descending branch (LAD) was ligatured. The rats were euthanized at 7 or 28 days after MI, and the tissues were harvested for specific protocols. The rats were divided into four groups with five rats in each group: group 1: Ad-NC-Antagomir-17 for 7 days, group 2: Ad-Antagomir-17 for 7 days, group 3: Ad-NC-Antagomir-17 for 28 days, and group 4: Ad-Antagomir-17 for 28 days. The sham groups were exposed to the surgery without AMI and continued to be fed for 7 or 28 days. Cardiac function was evaluated by echocardiography before surgery and was re-evaluated at 7 or 28 days after MI. For histology, the tissues were perfusion-fixed with buffered formalin phosphate, and the hearts were harvested and processed. For real-time RT-PCR and western blot, the tissues were freshly removed and snap-frozen, as described above.

### Morphometric analysis

At 7 or 28 days after the operation, the hearts were harvested, fixed in 4% paraformaldehyde, and then embedded in paraffin. For morphologic analysis, cardiac tissue at the infarction border area was removed. The infarction areas were determined by HE staining. Masson staining was used to examine collagen fiber areas. Apoptosis of cardiac tissue was determined by TUNEL staining; data are presented as percentages of total cells that are positive for apoptosis within a given area. CD31 staining was used to evaluate microcirculation formation; the perimeters of the areas positive for CD31 were measured, and the percentages of the total perimeters were also calculated.

### Statistical analysis

Data are presented as means ± SEM. All values were analyzed using Student’s *t* test for comparisons between two groups. A *p* value of < 0.05 was considered statistically significant.

## Results

### Inhibition of miR-17-5p attenuates HUVEC apoptosis induced by culturing in ECM without FBS

**Fig. 1:**
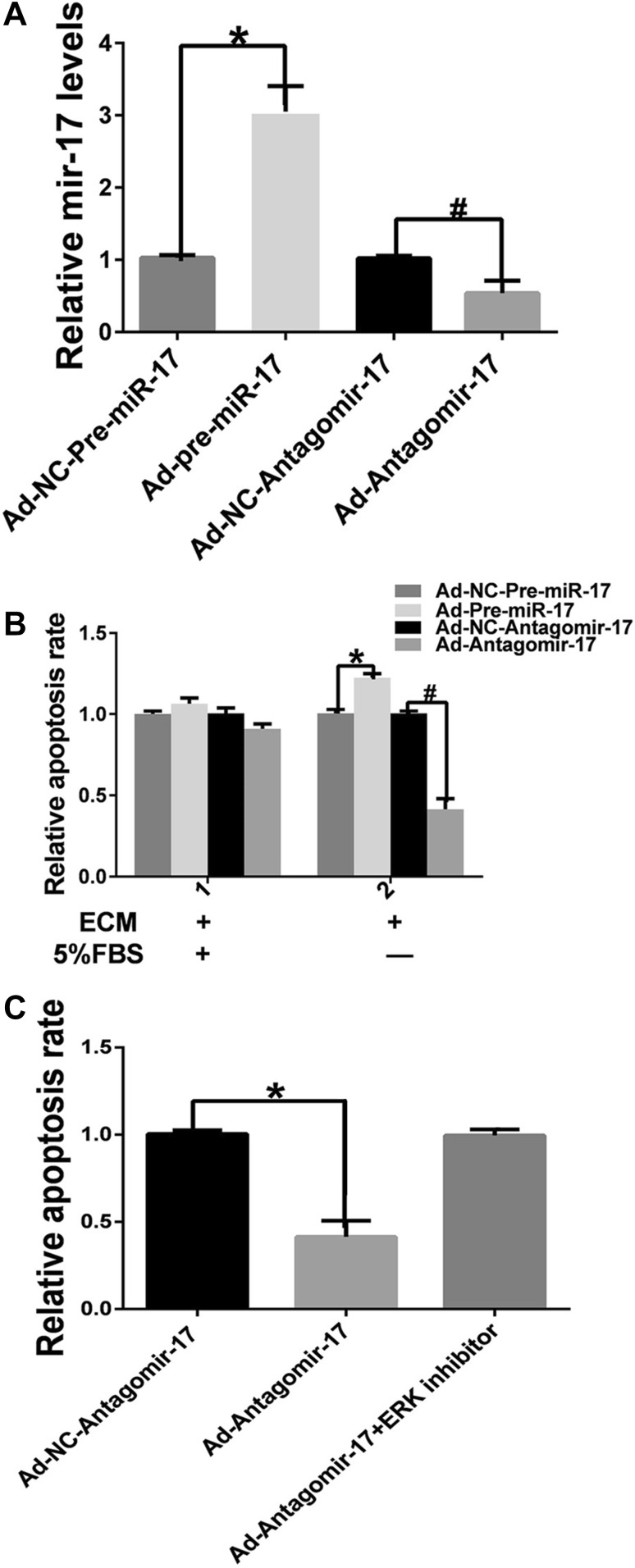
MicroRNA-17-5p inhibition suppressed HUVEC apoptosis. **a** HUVECs were infected with adenovirus for 4 h and cultured with ECM containing 5% FBS for 72 h. Total mRNA was extracted, and the miR-17-5p level was detected by real-time RT-PCR (*n* = 3). **b** HUVECs infected with adenoviruses were divided into two parts; one part underwent apoptosis induction via incubation with ECM lacking FBS for 24 h, and the other part was cultured with ECM containing 5% FBS. Quantification of cellular apoptosis was performed with an Annexin V-7AAD/PI assay. **c** Apoptosis of HUVECs pretreated with the ERK inhibitor PD98059 (10 µM) was evaluated by an Annexin V-7AAD/PI assay (*p < 0.05 vs. Ad-NC-Pre-miR-17, ^#^*p* < 0.05 vs. Ad-NC-Antagomir-17, *n* = 3)

After infection with adenoviruses, the level of miR-17-5p in the HUVECs was determined by real-time RT-PCR to evaluate the transfection efficiency. The miR-17-5p level was downregulated by 23% in the Ad-Antagomir-17 group and was up-regulated by 2.7-fold in the Ad-Pre-miR-17 group. Mir-17-5p silencing inhibited apoptosis in apoptosis-induced HUVECs. Inversely, up-regulation of miR-17-5p promoted apoptosis in apoptosis-induced HUVECs. However, in HUVECs cultured with ECM containing 5% FBS, the apoptosis ratios were low in both the miR-17-5p overexpression and miR-17-5p silencing HUVECs. To further assess whether apoptosis inhibition by miR-17-5p depends on the expression of ERK in HUVECs, the inhibitory effect of the ERK inhibitor PD098059 was evaluated. Treatment with PD98059 did not inhibit HUVEC apoptosis after miR-17-5p silencing in vitro (Fig. 1).

### Mir-17-5p silencing stimulates ERK-signaling pathway

**Fig. 2:**
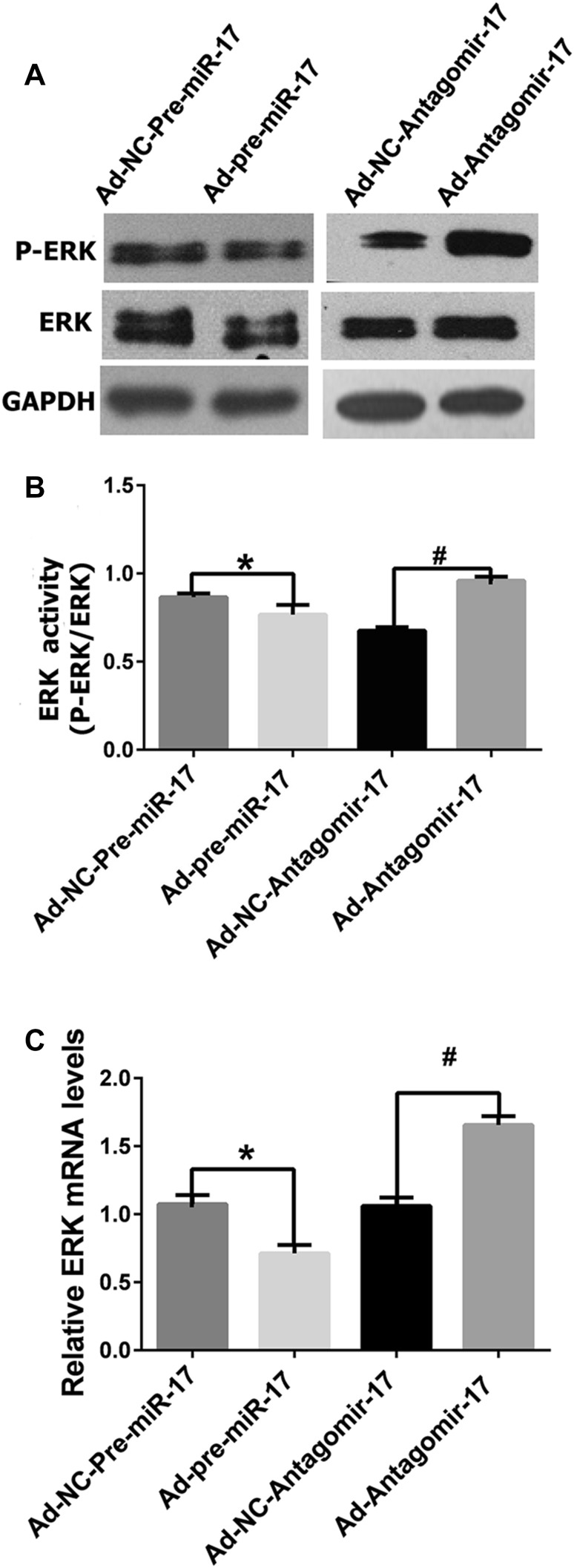
Effects of miR-17-5p on the ERK pathway. **a** HUVECs were infected with adenovirus for 4 h and cultivated with ECM containing 5% FBS for 72 h. Then, they were cultivated with ECM without FBS for another 24 h to induce apoptosis. The cells were collected, and the protein levels of ERK and P-ERK were measured. **b** Protein levels of ERK, P-ERK in the HUVECs mentioned above were examined by western blot, and ERK activity was evaluated by calculating P-ERK/ERK. **c** mRNA levels of ERK were examined by PCR (*p < 0.05 vs. Ad-NC-Pre-miR-17, ^#^*p* < 0.05 vs. Ad-NC-Antagomir-17, *n* = 3)

To further explore the mechanism of miR-17-5p on apoptosis, several key apoptosis pathways were examined. In apoptosis-induced HUVECs, the ERK pathway was stimulated by miR-17-5p silencing, as indicated by increased mRNA level of ERK and protein levels of ERK and phosphorylated ERK (P-ERK). ERK activity was evaluated by calculating P-ERK/ERK (Fig. 2).

### Mir-17-5p silencing inhibits apoptosis through the ERK signaling pathway

**Fig. 3:**
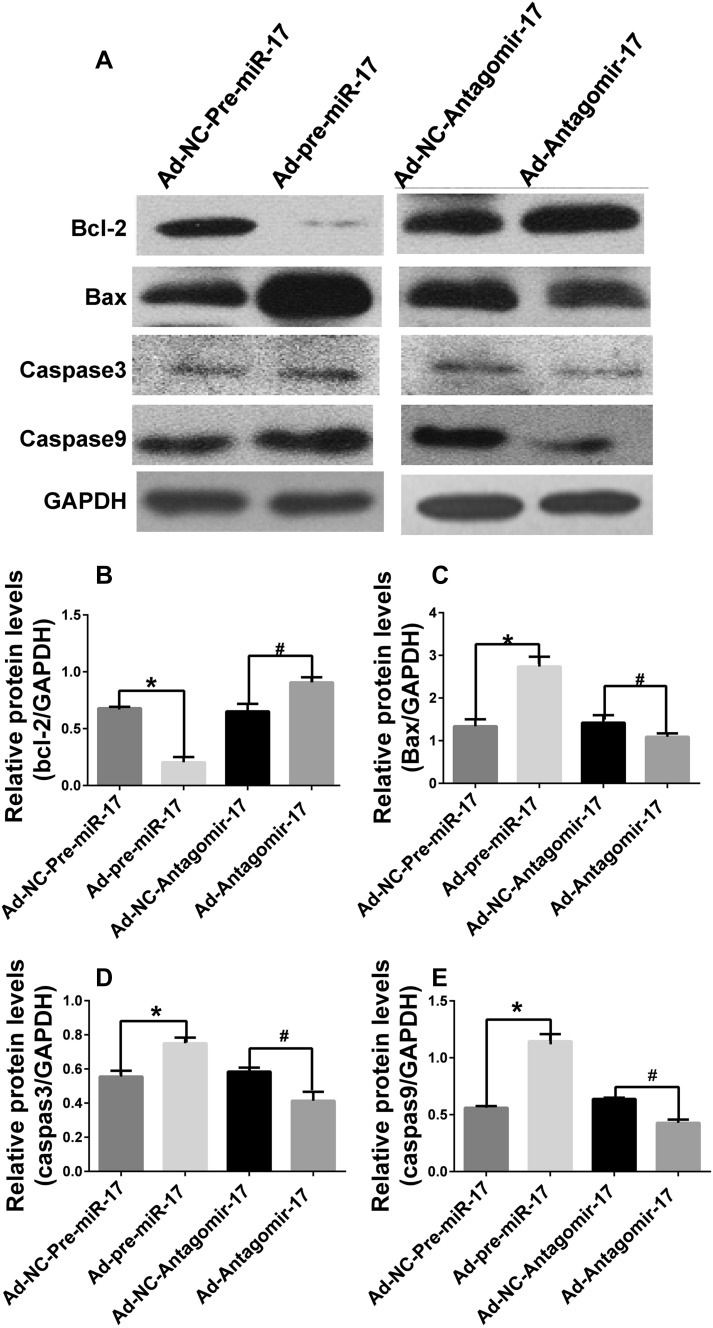
Effect of miR-17-5p on apoptosis-related protein levels. **a** HUVECs were infected with adenovirus for 4 h and cultivated with ECM containing 5% FBS for 72 h. Then, they were cultivated with ECM without FBS for another 24 h to induce apoptosis. The cells were collected, and the protein levels of apoptosis-related protein were measured. **b** Protein levels of anti-apoptosis protein bcl-2 were measured. **c** Levels of apoptosis protein Bax was measured. **d** Relative protein levels of apoptosis protein caspas3. **e** Relative protein levels of apoptosis protein caspas9 (*p < 0.05 vs. Ad-NC-Pre-miR-17, ^#^*p* < 0.05 vs. Ad-NC-Antagomir-17, *n* = 3)

To further investigate the effect of miR-17-5p on apoptosis via ERK pathway, several key downstream proteins were examined. In apoptosis-induced HUVECs, the protein level of the anti-apoptosis protein bcl-2 increased, and the levels of apoptosis proteins, including bax, caspase 9, and caspase 3, were reduced by miR-17-5p silencing; the opposite effect was observed with miR-17-5p overexpression (Fig. 3).

### Inhibition of miR-17-5p inhibits apoptosis of rat heart after AMI via the ERK pathway

**Fig. 4:**
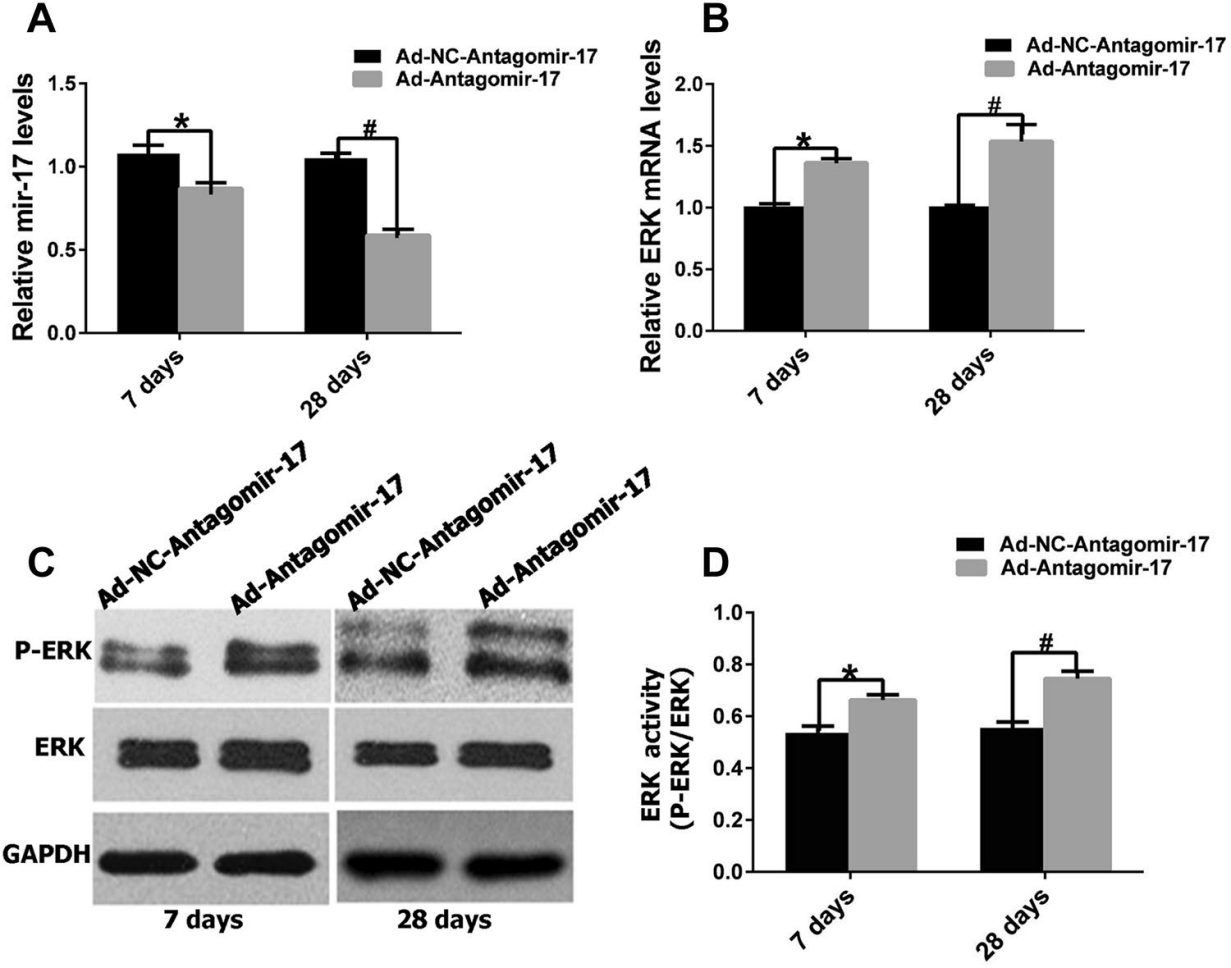
Effects of miR-17-5p inhibition on the ERK pathway in injured cardiac tissue. After 7 or 28 days AMI, **a** mRNA expression of miR-17 was analyzed by real-time RT-PCR (*n* = 3, each group contains 5 hearts). **b** mRNA expression of ERK was analyzed by real-time RT-PCR (*n* = 3, each group contains 5 hearts). **c** Protein expression of ERK, P-ERK were examined by western blot. **d** ERK protein activity was evaluated by P-ERK/ERK (*n* = 3, each group contains 5 hearts) (*p < 0.05 vs. Ad-NC-Antagomir-17, ^#^*p* < 0.05 vs. Ad-NC-Antagomir-17, *n* = 3)

**Fig. 5:**
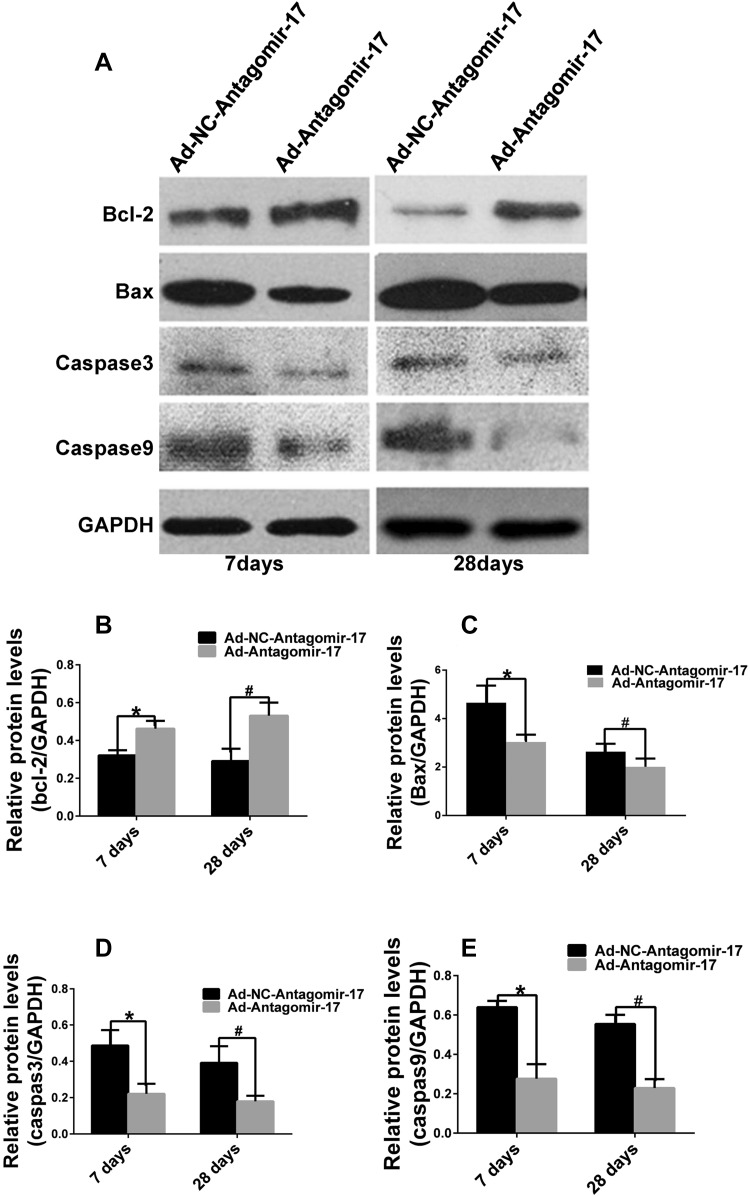
Effects of miR-17-5p inhibition on apoptosis-related proteins in injured cardiac tissue. After 7 or 28 days AMI, **a** Western blot was used to detected apoptosis-related proteins. **b** Protein expression of anti-apoptosis protein bcl-2 was examined by western blot. **c** Levels of apoptosis protein Bax was measured by western blot. **d** Protein expression of apoptosis protein caspas3 was examined by western blot. **e** Relative protein levels of apoptosis protein caspas9 (*n* = 3, each group contains 5 hearts) (*p < 0.05 vs. Ad-NC-Antagomir-17, ^#^*p* < 0.05 vs. Ad-NC-Antagomir-17, *n* = 3)

As shown in Figs. 4 and 5, miR-17-5p levels were decreased by 19.7 and 41.2%, respectively, in hearts from rats in the Ad-Antagomir-17 group and the Ad-NC-Antagomir-17 group at 7 or 28 days after the establishment of MI. Subsequent analysis of the protein expression levels of the apoptosis proteins mentioned above demonstrated a blunted response, and the anti-apoptosis protein bcl-2 demonstrated an intense response following miR-17-5p silencing compared to the control group at 7 or 28 days. This anti-apoptosis effect of miR-17-5p silencing was accompanied by increased mRNA expression levels of ERK and P-ERK, as observed by western blot.

### Effect of miR-17-5p on histology of rat heart after MI

**Fig. 6:**
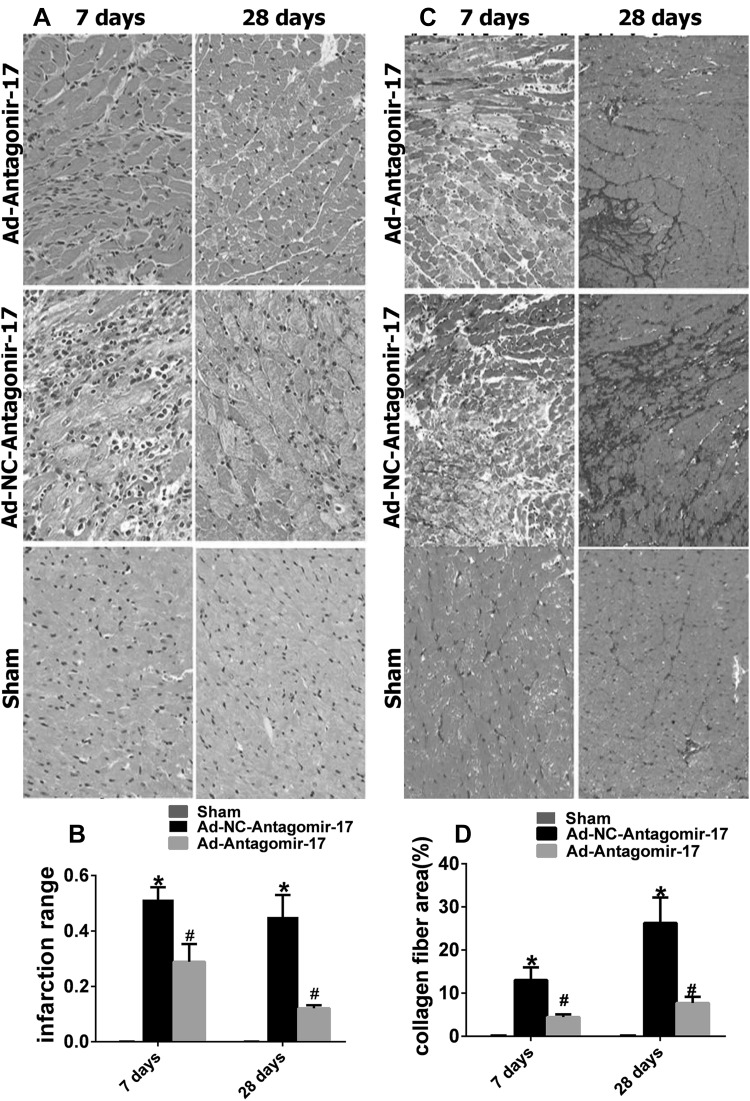
Effect of miR-17-5p inhibition on the histopathology in heart tissue after myocardial infarction at 7 or 28 days after the induction of AMI. **a, b** Representative hematoxylin and eosin-stained cardiac tissues from each experimental group (*n* = 5, light microscope, ×400). **C, d** Representative Masson trichrome-stained heart tissues. Collagen is stained blue (*n* = 5, light microscope, ×200) (^#^*p* < 0.05 vs. *Ad-NC-Antagomir-17)

After 7- or 28-day MI, as shown in Fig. 6, HE staining showed that infarction areas were decreased by 43.7 and 73.2% in the Ad-Antagomir-17 group and Ad-NC-Antagomir-17 group, respectively (*p* < 0.05). Masson staining also indicated that areas of collagen fibers were reduced by 53.3 and 61.1% after miR-17-5p silencing in the Ad-Antagomir-17 group and Ad-NC-Antagomir-17 group, respectively (*p* < 0.05). Therefore, downregulation of miR-17-5p could protect cardiac structure of rat heart after AMI.

### Effect of miR-17-5p on immunohistochemistry of rat heart after MI

**Fig. 7:**
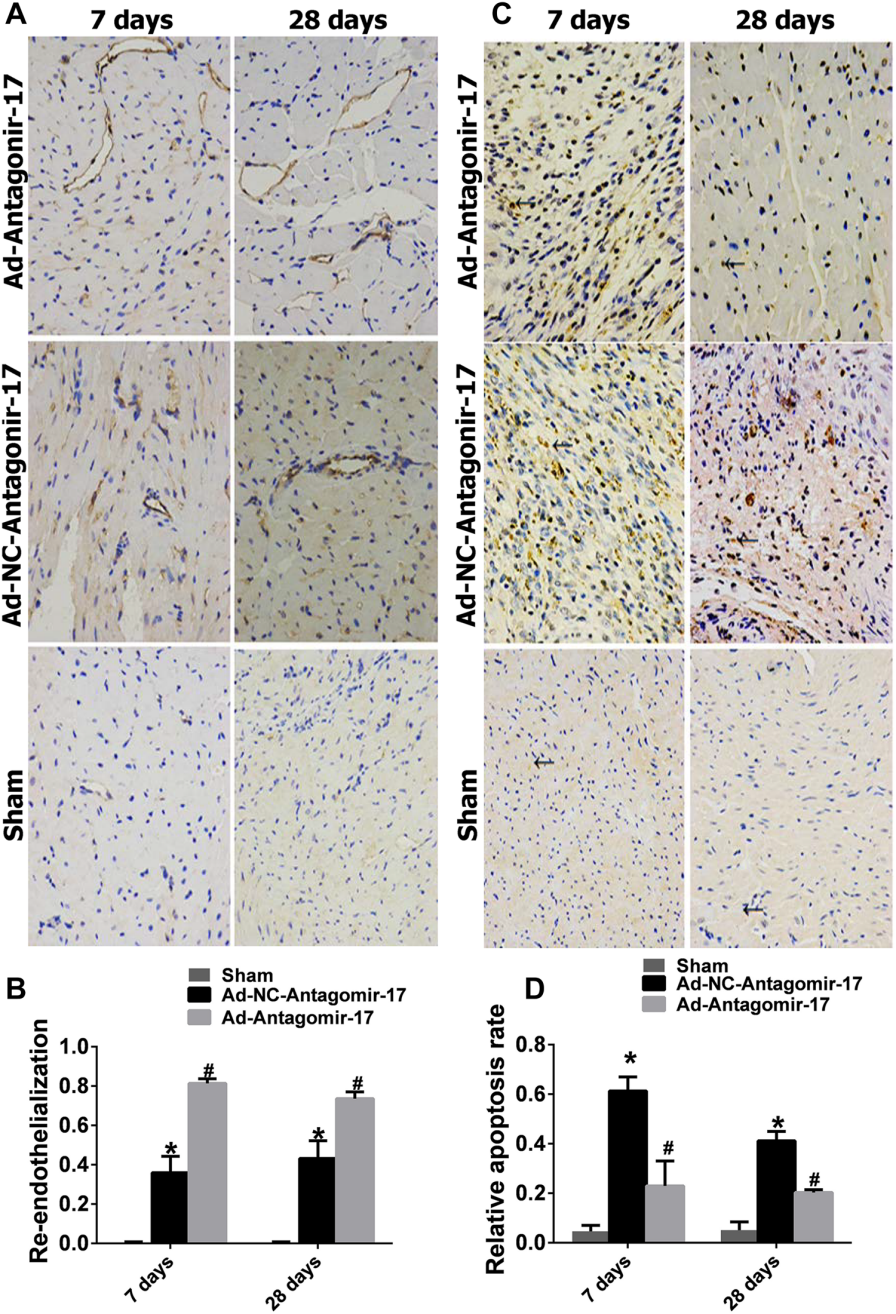
Effect of miR-17-5p inhibition on the histopathology in heart tissue after myocardial infarction at 7 or 28 days after the induction of AMI. **a, b** Representative cardiac sections with immunohistochemical staining for CD31. Black arrows indicate areas of endothelial recovery, which are stained dark brown (*n* = 5, light microscope, ×400). **c, d** Representative TUNEL-stained cardiac sections. Black arrows indicate positive cells, which are stained dark brown (*n* = 5, light microscope, ×400) (^#^*p* < 0.05 vs. *Ad-NC-Antagomir-17)

After 7- or 28-day MI, as shown in Fig. 7, apoptosis of cardiac tissue cells was examined by TUNEL staining, which showed that downregulation of miR-17-5p inhibited the apoptosis of cardiac tissue cells compared to the Ad-NC-Antagomir-17 group (0.23 ± 0.101 vs. 0.61 ± 0.056 at 7 days after MI, 0.20 ± 0.0108 vs. 0.41 ± 0.038 at 28 days after I, *p* < 0.05). CD31, a marker of microcirculation formation, was stained and also indicated that miR-17-5p silencing promotes endothelial growth after MI compared to the Ad-NC-Antagomir-17 group (0.814 ± 0.047 vs. 0.360 ± 0.166 at 7 days after MI, 0.736 ± 0.069 vs. 0.432 ± 0.181 at 28 days after MI, *p* < 0.05). Thus, miR-17 silence could promote microcirculation of rat heart after AMI.

### Effect of miR-17-5p on rat heart function after MI

At 7 or 28 days after MI, cardiac function was evaluated morphologically and quantitatively. Many index were used to determine the cardiac function, like ejection fractions (EF), fractional shortening (FS), end diastolic volume (EDV), End systolic volume (ESV), left ventricular internal dimension diastole (LVIDD), left ventricular internal dimension systole (LVIDS). As shown in Table 1, echocardiography revealed that downregulation of miR-17-5p enhanced heart function. The data mean percent decrease compared with heart function before AMI.

**Table 1:** Effect of miR-17-5p inhibition on heart function after myocardial infarction

	Ad-NC-Antagomir-17 28 days	Ad-Antagomir-17 28 days	Ad-NC-Antagomir-17 7 days	Ad-Antagomir-17 7 days
EF	0.3723 ± 0.05393	0.06021 ± 0.02443*	0.3406 ± 0.03382	0.08890 ± 0.02123*
FS	− 0.5453 ± 0.1118	− 0.1298 ± 0.05233*	− 0.5048 ± 0.07902	− 0.1706 ± 0.04511*
EDV	2.879 ± 0.1945	1.022 ± 0.2184*	2.016 ± 0.3738	0.3111 ± 0.05300*
ESV	28.00 ± 3.372	6.000 ± 2.517*	24.33 ± 3.528	8.444 ± 2.764*
LVIDD	0.9624 ± 0.2147	0.2828 ± 0.05307*	0.8422 ± 0.1362	0.2681 ± 0.1519*
LVIDS	1.274 ± 0.2665	0.4556 ± 0.1056*	2.924 ± 0.5913	0.8479 ± 0.3661*

*P < 0.05 which represent that the indicators (EF, FS, EDV, ESV, LVIDD and LVIDS) had significant differences between downregulation group (Ad-Aatagomir-17) and control group (Ad-NC-Aatagomir-17)

Heart function was evaluated by echocardiography before the models were built and at 7 and 28 days later. The percentages of heart function decrease in each group were calculated (*p < 0.05 vs. Ad-NC-Antagomir-17).

## Discussion

Many miRNAs have been reported to regulate senescence positively or negatively, but most studies have focused on the effect of miRNAs on the senescence of tumor cells at cellular level, or investigated the role of miRNAs in lower organisms (Du et al. 2015). Uniquely, our study systematically describes the functions of one specific miRNA, miR-17. MicroRNA-17 has been investigated in many fields, such as B-cell lymphoma (Lu et al. 2011), acute organ-specific autoimmune disease (Battistella et al. 2015), glucocorticoid-induced osteoclast differentiation and function (de Kouchkovsky et al. 2013), and so on. It is involved in angiopoiesis (Shi et al. 2014), apoptosis (Shan et al. 2013), and proliferation (Honegger et al. 2015) in tumor tissues. However, the relationship between miR-17 and cardiovascular disease and the mechanisms underlying that relationship are still unknown. It is reported that miR-17 is sufficient to induce cardiomyocyte proliferation, but have nothing to do with cardiomyocyte apoptosis (Chen et al. 2013). However, this study focused on the effect of miR-17 on the apoptosis of ECs induced by serum deprivation. Our results indicated that miR-17-5p silencing had a greater apoptosis inhibition effect on ECs under serum-deprived conditions and had a nearly insignificant effect on ECs under normal conditions. The apoptosis rate is low under normal circumstances; thus, the apoptosis mechanism could not be triggered in the cells grown under normal conditions. These results show that miR-17-5p plays an important role in making life or death choices in ECs when apoptosis is triggered.

One major question being investigated in this study was how miR-17-5p silencing decreases the apoptotic rate of ECs in response to serum deprivation. Apoptosis can be triggered by extracellular stimuli via the death receptor and mitochondrial-mediated pathways (Scarabelli et al. 2002). The ERK pathway plays an important role in several cellular processes, such as cell survival, cell proliferation and apoptosis. It has been reported that activated ERK is responsible for high levels of cell growth in various cancers (Dhillon et al. 2007; Yang et al. 2013). Meanwhile, inhibition of activated ERK is associated with apoptosis (Salminen et al. 2010). Inhibition of miR-17-5p promotes the expression of activated ERK both in vivo and in vitro. Thus, we hypothesized that downregulation of miR-17-5p would decrease the apoptotic rate of endothelial cells both in vivo and in vitro. Proteins of the bcl-2 family play a pivotal role in apoptosis (Baell and Huang 2002; Goodsell 2002). BCL-2 interacting mediator of cell death (BIM) can be phosphorylated by members of mitogen activated protein kinase family. Extracellular signal-regulated kinase 1/2 (ERK1/2)-mediated phosphorylation stimulates BIM degradation via the proteasome (Luciano et al. 2003; Kennedy et al. 2017). Bcl-2 is thought to be an anti-apoptotic protein and can inhibit the activity of the caspase cascade after it is initiated by the mitochondrial release of cytochrome c (Rosse et al. 1998; Cao et al. 2015). In contrast, bax is thought to be a proapoptotic protein of the bcl-2 family (Sedlak et al. 1995; Kluck et al. 1997), which can result in permeabilization of mitochondria (Rosse et al. 1998; Cao et al. 2015). We also detected the protein levels of caspase 3 and caspase 9, which, when in their active state, play a crucial role in the final step of apoptosis (Shebaby et al. 2014). Our research found that downregulation of miR-17 increased the protein level of bcl-2, while levels of the bax/caspase 3/caspase 9 proteins were decreased, which is consistent with inhibition of apoptosis.

Heart failure after myocardial infarction is a clinically common phenomenon. There is high morbidity and mortality following myocardial infarction, because the remaining heart tissue has to maintain adequate cardiac output, similar to the current management of congestive heart failure (Kanashiro-Takeuchi et al. 2015). Our data showed that have shown that heart failure after myocardial infarction is closely related to the microcirculation damage of the heart (Teunissen et al. 2015). Heart function can be protected if microcirculation is improved after myocardial infarction, which would prevent symptoms of heart failure. Research has verified that inhibiting the expression of miR-17-5p can prevent endothelial cells from undergoing apoptosis and can also promote tube formation by vessel endothelial cells. Therefore, MiR-17-5p silencing protects heart function after AMI by improving the microcirculation of the heart tissue, which is through decreasing the rate of apoptosis and repairing vascular injury. The effect of miR-17 on the heart is still not entirely clear, and many more potential mechanisms underlying this effect must be investigated.

## Conclusion

Collectively, this study provided novel insights into the mechanism of miR-17-5p regulating endothelium apoptosis. Actually, apoptosis was inhibited by the downregulation of miR-17-5p through activation of the ERK pathway. MiR-17-5p silencing protects heart function after AMI by improving the microcirculation of the heart tissue by decreasing the rate of apoptosis and repairing vascular injury.
